# Supplementary material for: The Synthesis and Evaluation of Diethyl Benzylphosphonates as Potential Antimicrobial Agents
Source: Molecules. 2022 Oct 13;27(20):6865. doi: 10.3390/molecules27206865 (PMC9612312; doi:10.3390/molecules27206865)
Supplement: Supplementary file 1 [file molecules-27-06865-s001.zip › molecules-1962756-supplementary.pdf]

Supplementary Materials

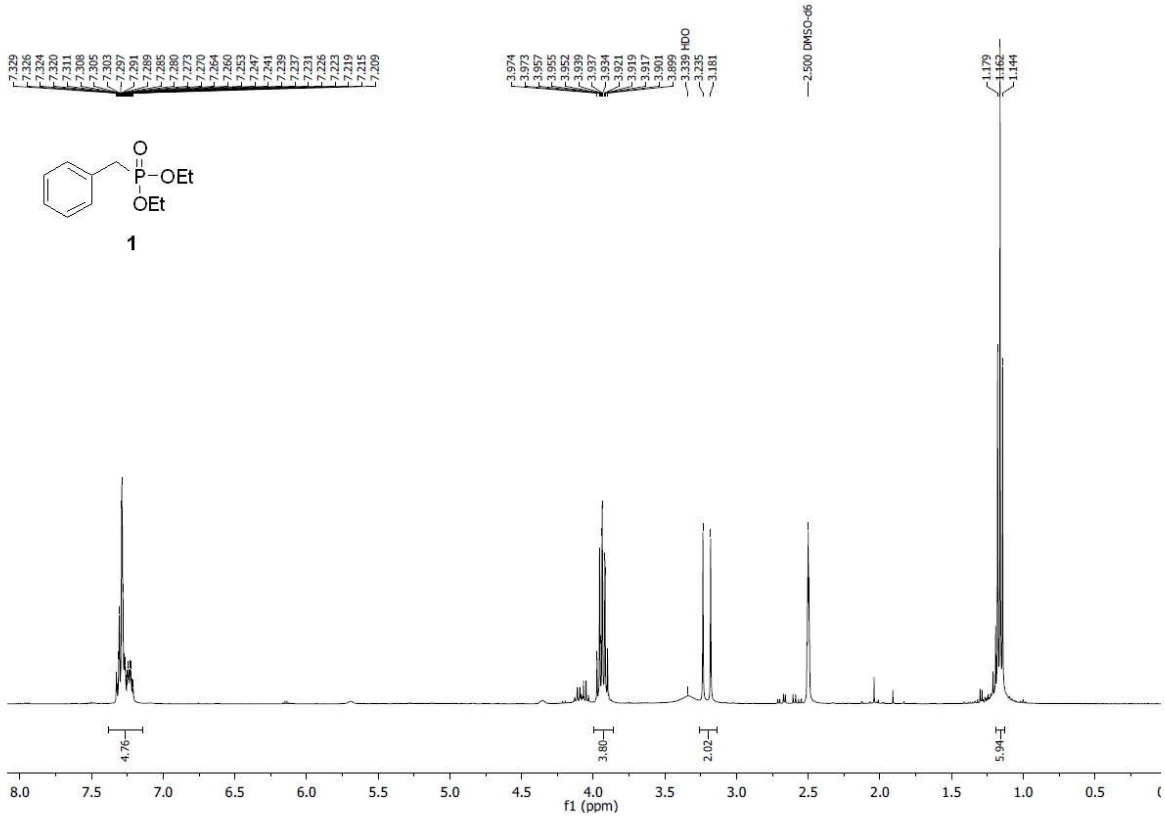

Figure S1. ^{1}H NMR (400 MHz, DMSO-d6) of diethyl benzylphosphonate (**1**).

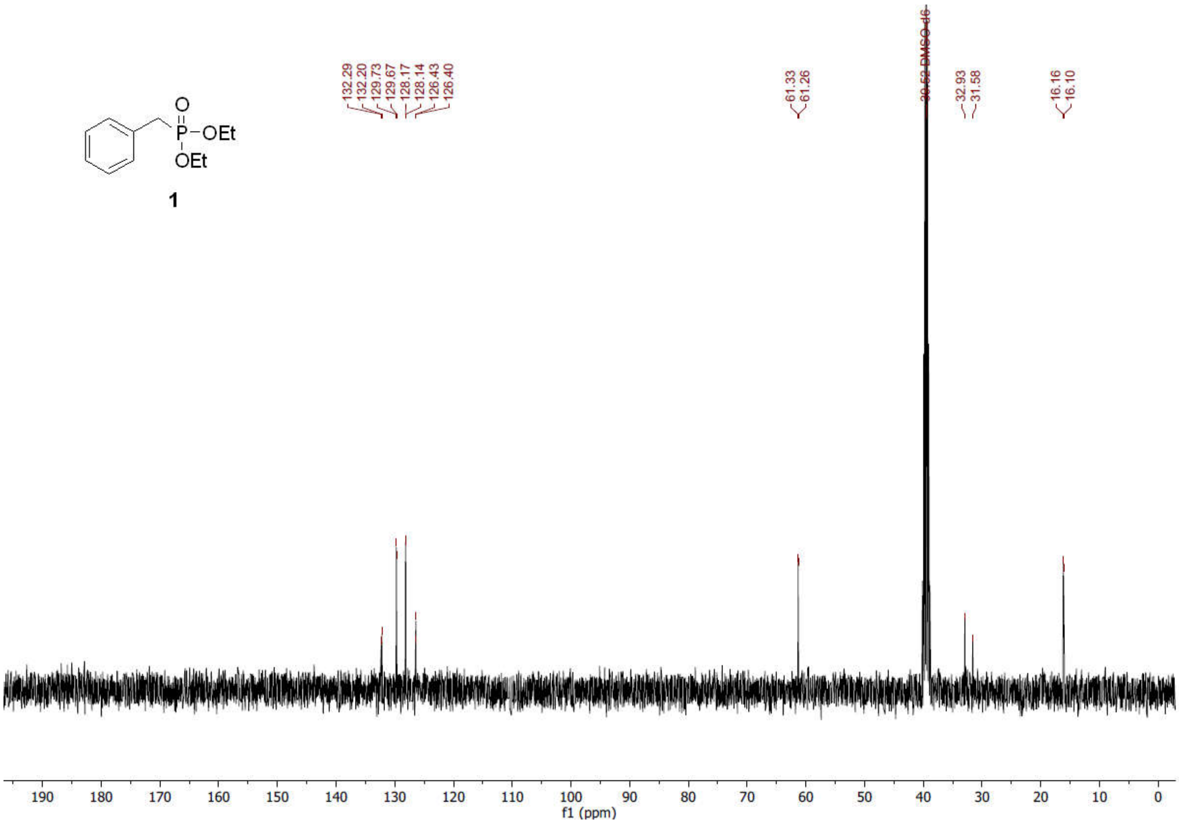

Figure S2. ^{13}C NMR (400 MHz, DMSO-d6) of diethyl benzylphosphonate (**1**).

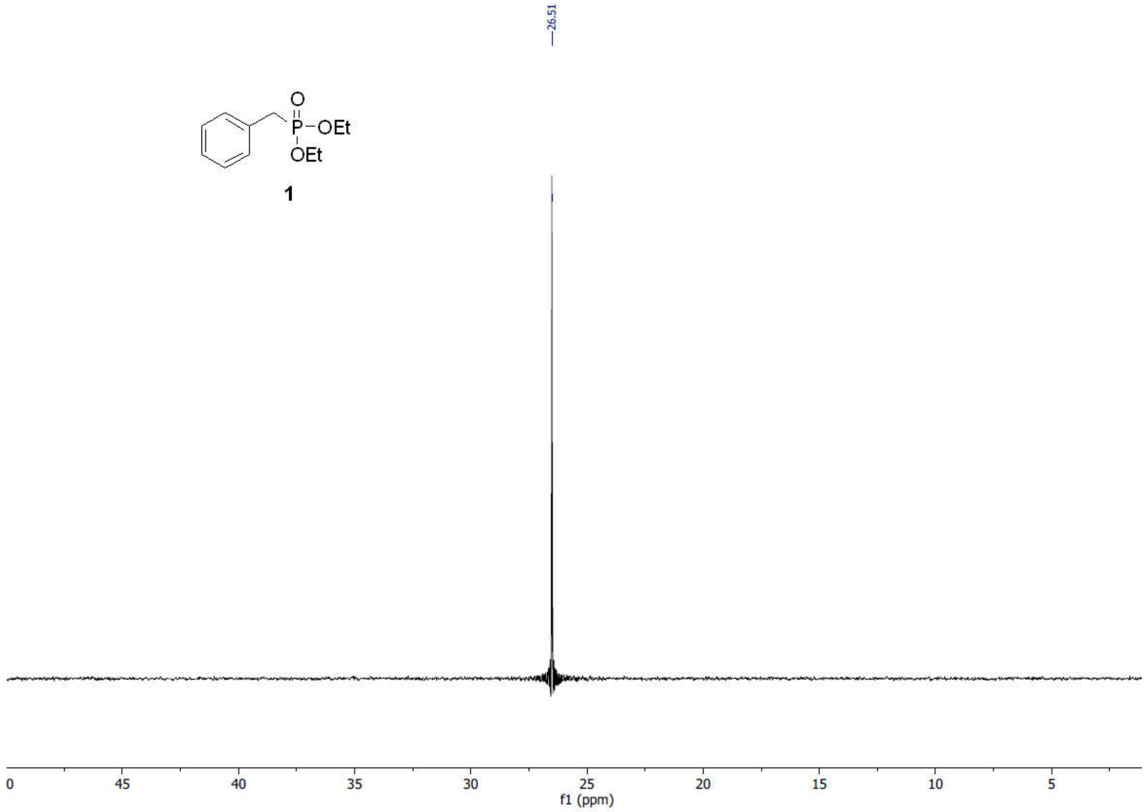

Figure S3. ^{31}P NMR (400 MHz, DMSO-d6) of diethyl benzylphosphonate (**1**).

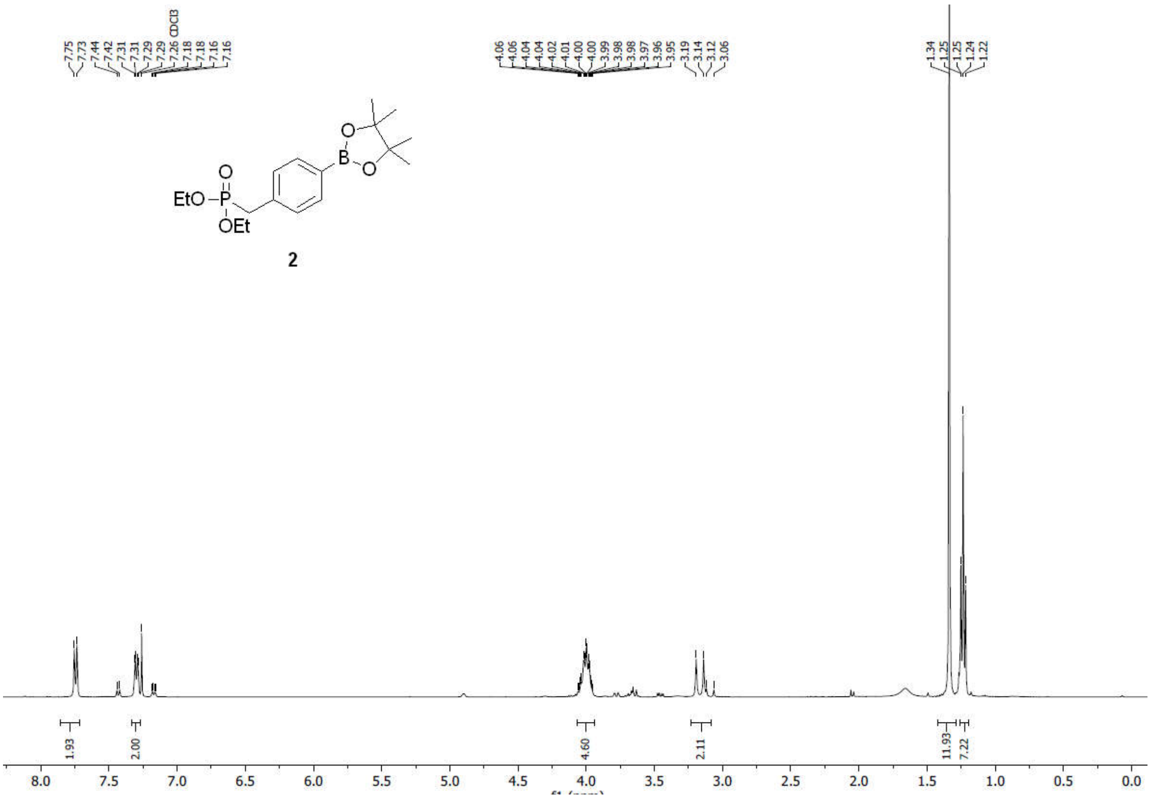

Figure S4. ^{1}H NMR (400 MHz, $CDCl_3$) of diethyl (4-(4,4,5,5-tetramethyl-1,3,2-dioxaborolan-2-yl)benzyl)phosphonate (**2**).

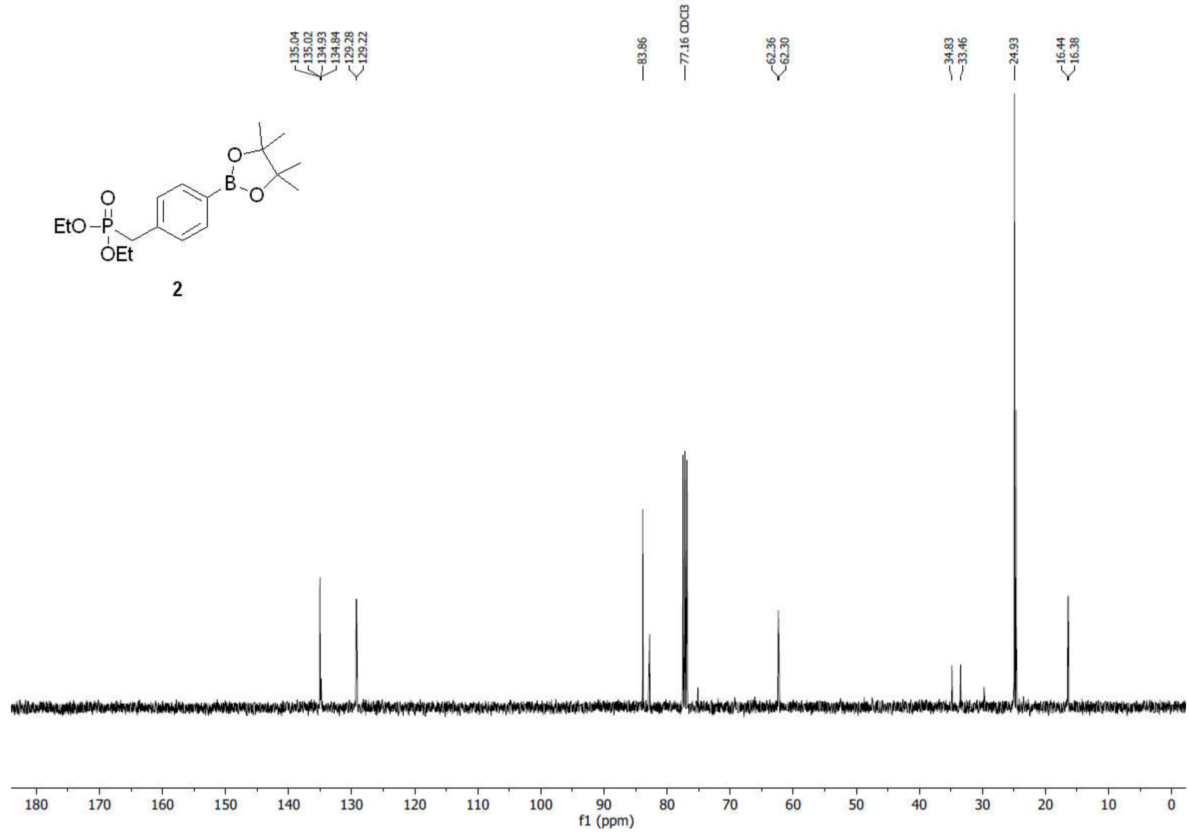

Figure S5. ^{13}C NMR (400 MHz, $CDCl_3$) of diethyl (4-(4,4,5,5-tetramethyl-1,3,2-dioxaborolan-2-yl)benzyl)phosphonate (**2**).

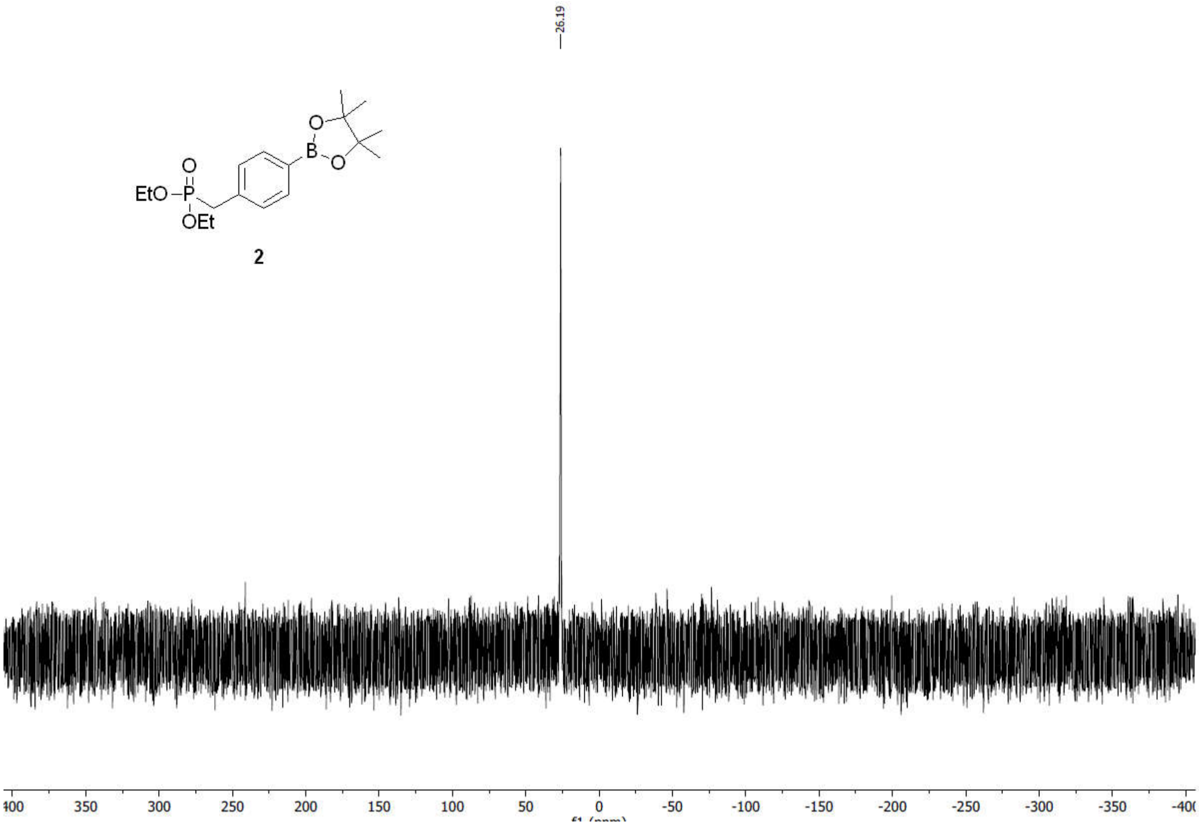

Figure S6. ^{31}P NMR (400 MHz, $CDCl_3$) of diethyl (4-(4,4,5,5-tetramethyl-1,3,2-dioxaborolan-2-yl)benzyl)phosphonate (**2**).

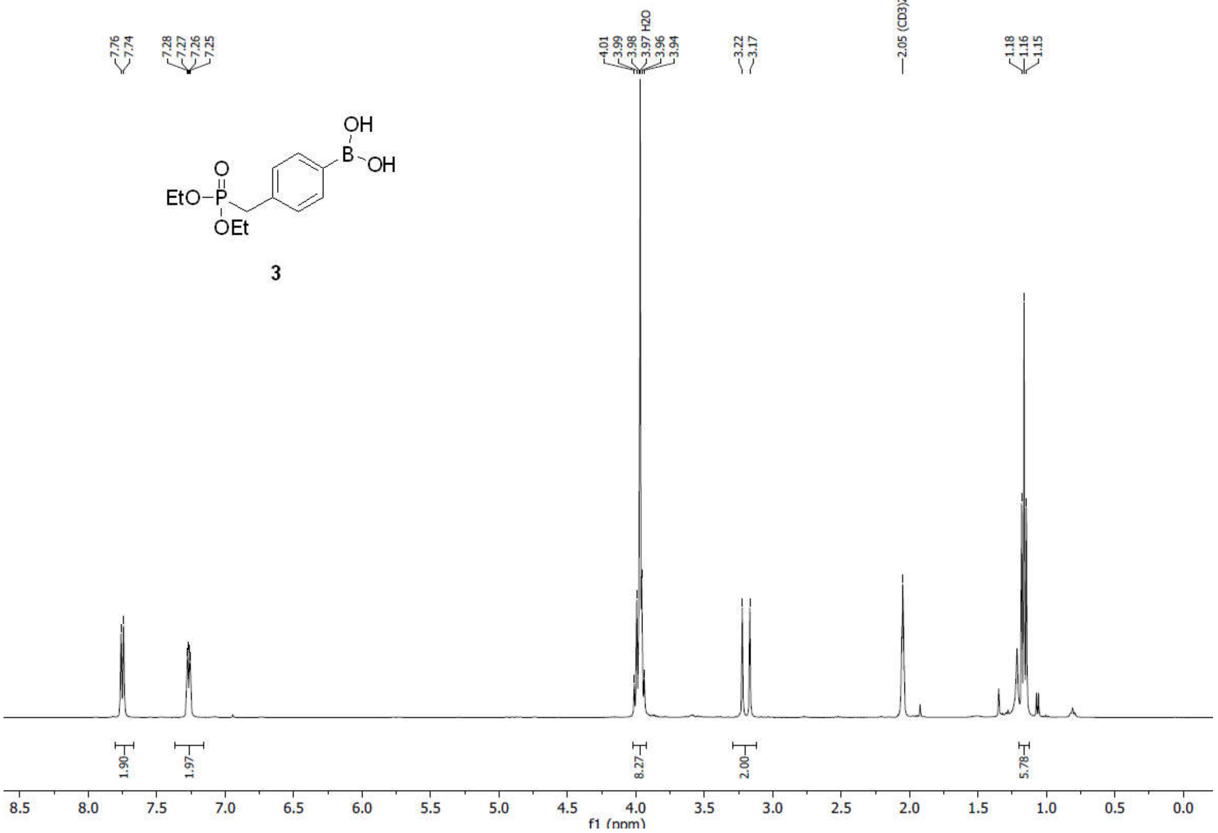

Figure S7. ^{1}H NMR (400 MHz, Acetone-d_6 + D_2O) of (4-((diethoxyphosphoryl)methyl)phenyl)boronic acid (**3**).

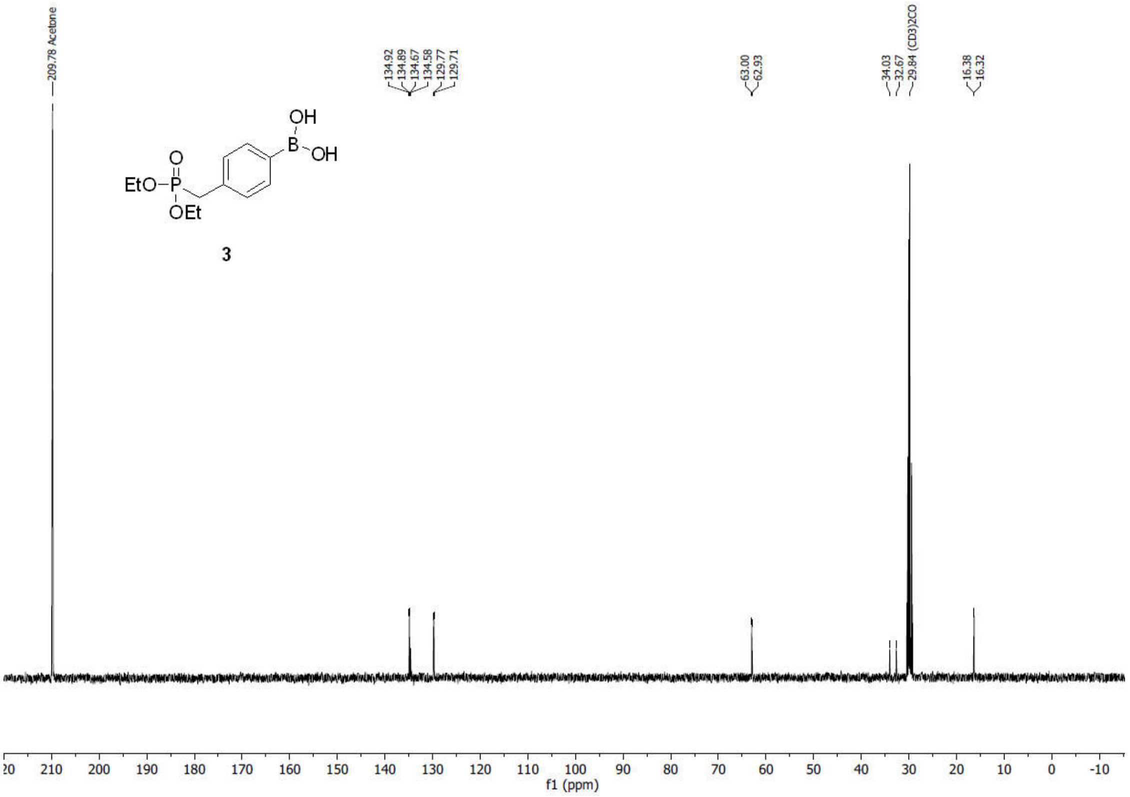

Figure S8. ^{13}C NMR (400 MHz, Acetone-d_6 + D_2O) of (4-((diethoxyphosphoryl)methyl)phenyl)boronic acid (**3**).

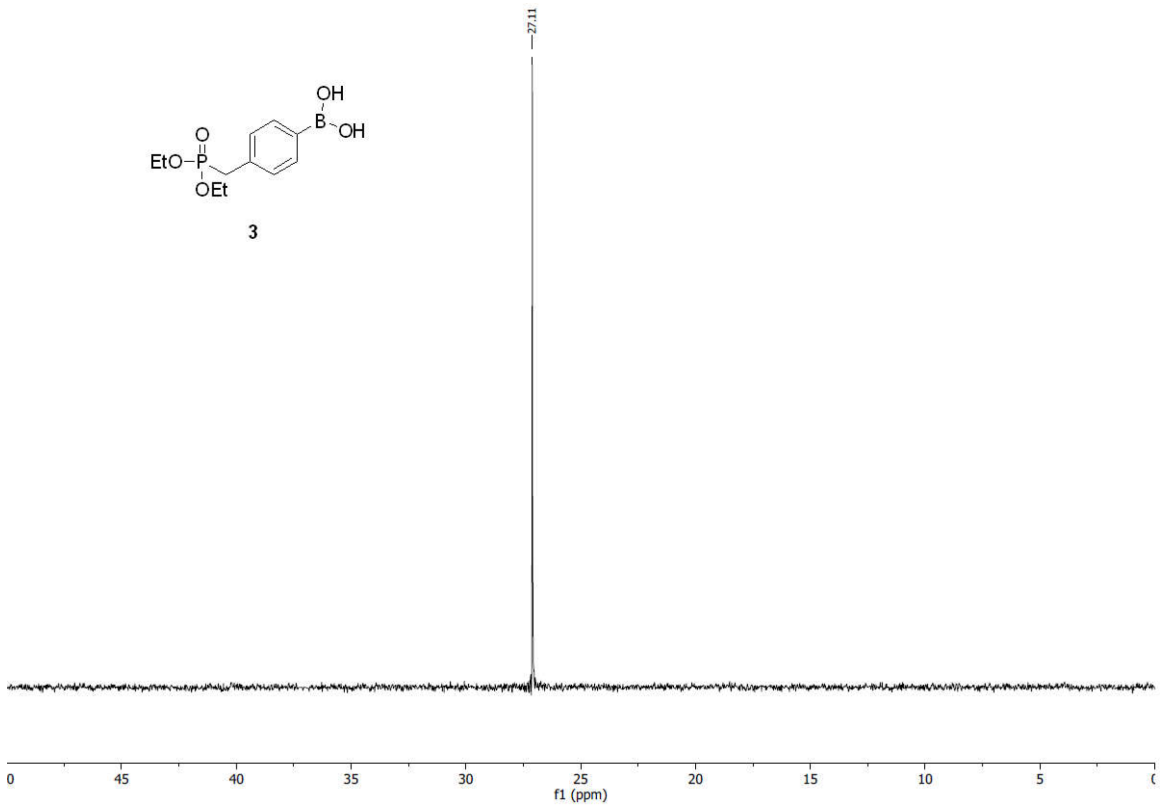

Figure S9. ^{31}P NMR (400 MHz, Acetone-d_6 + D_2O) of (4-((diethoxyphosphoryl)methyl)phenyl)boronic acid (**3**).

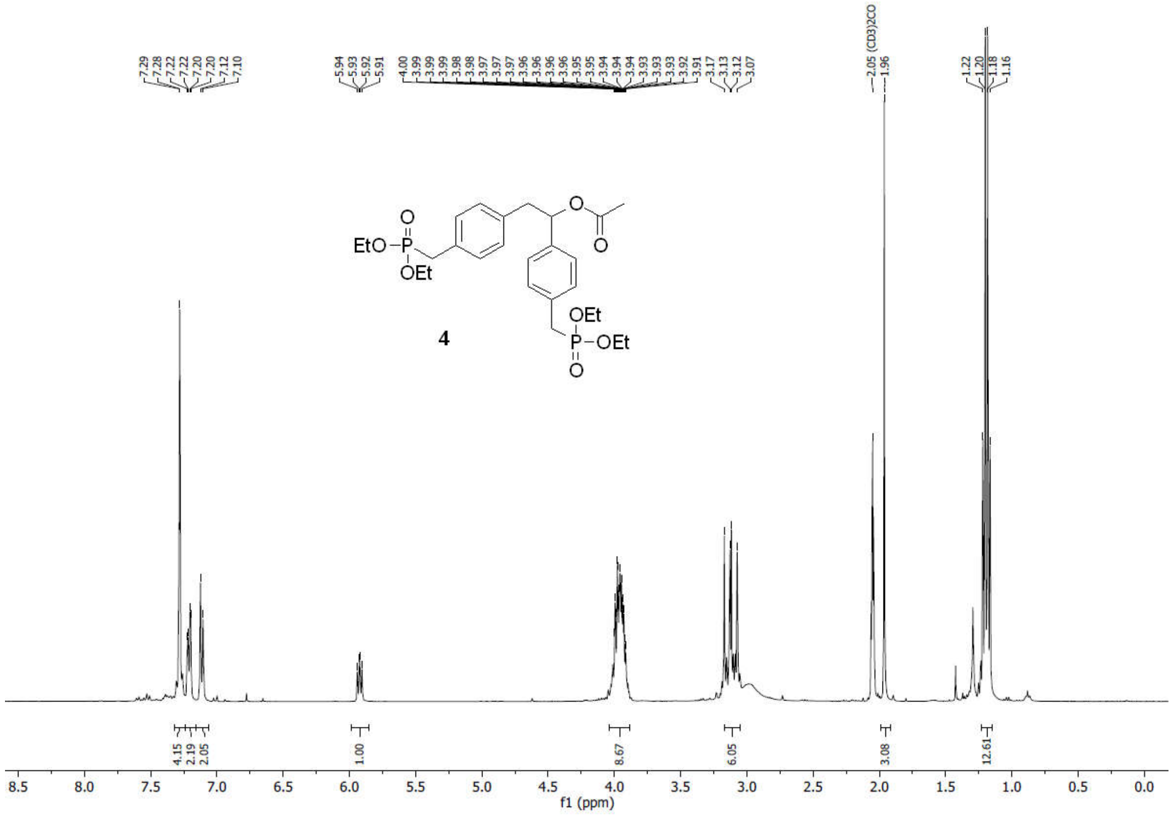

Figure S10. ^{1}H NMR (400 MHz, Acetone-d_6 + D_2O) of (1,2-bis(4-((diethoxyphosphoryl)methyl)phenyl) ethyl acetate (**4**).

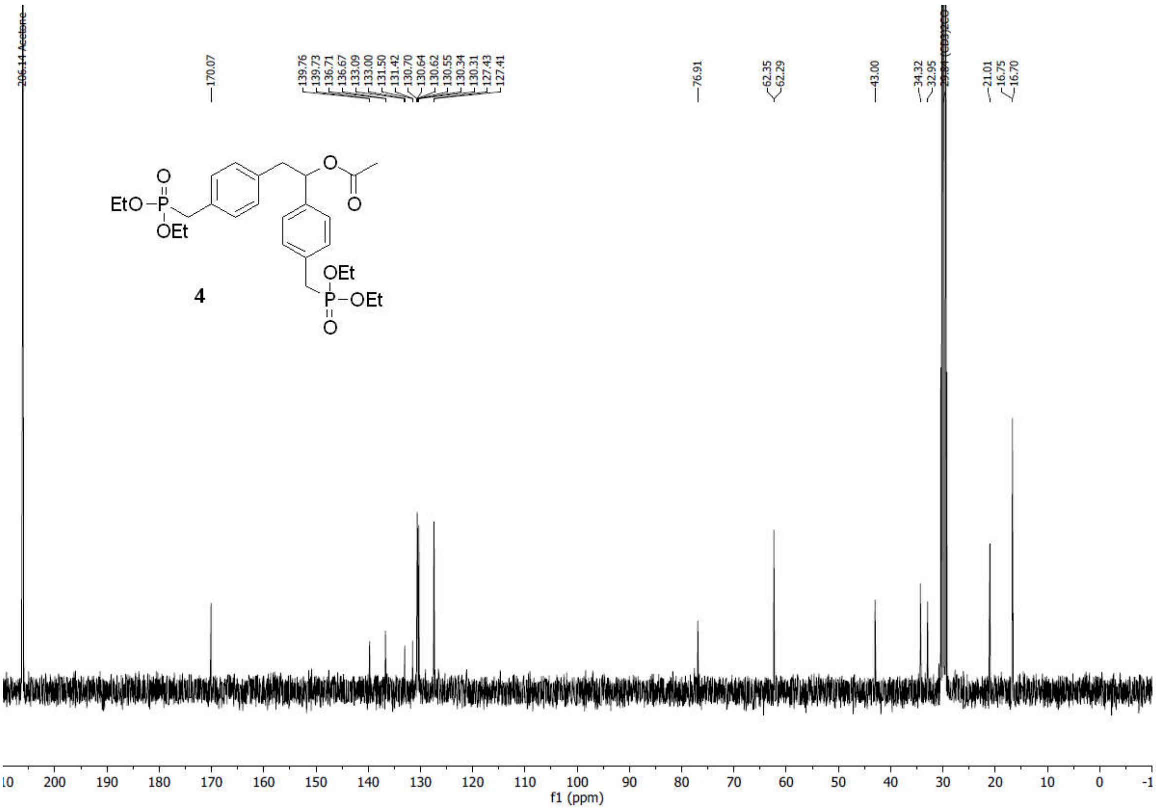

Figure S11. ^{13}C NMR (400 MHz, Acetone-d_6 + D_2O) of (1,2-bis(4-((diethoxyphosphoryl)methyl)phenyl) ethyl acetate (**4**).

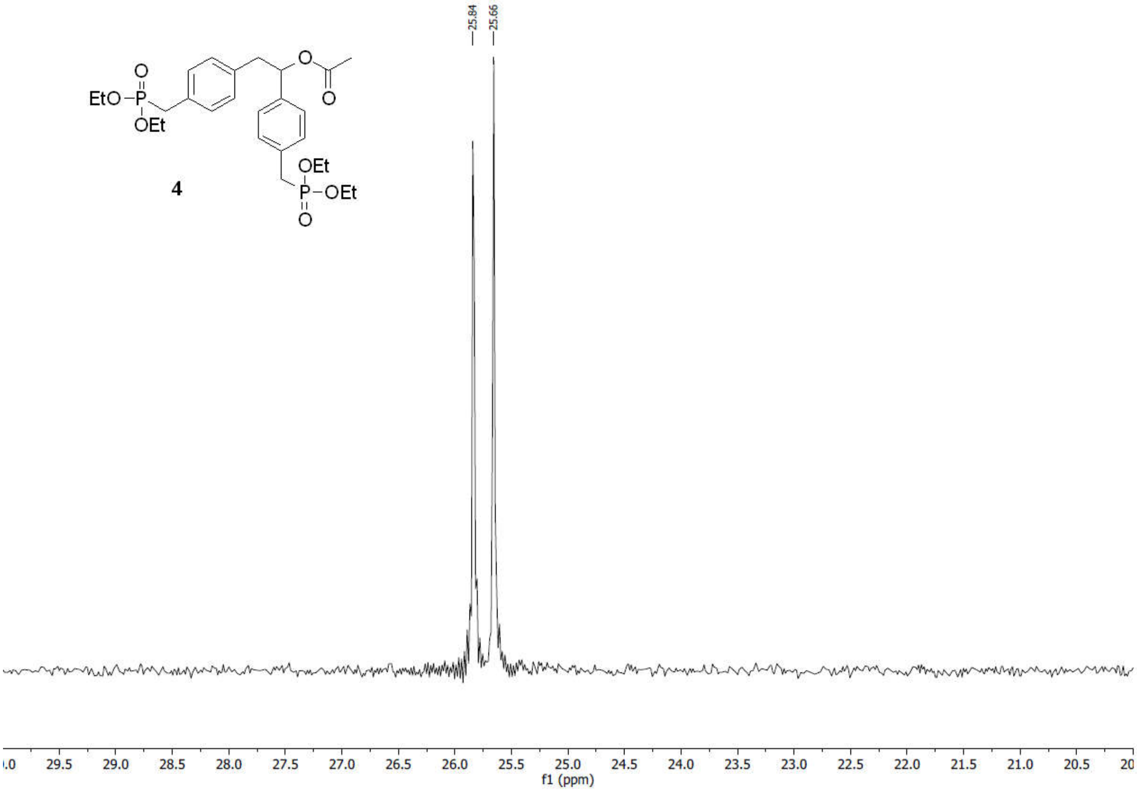

Figure S12. ^{31}P NMR (400 MHz, Acetone-d_6 + D_2O) of (1,2-bis(4-((diethoxyphosphoryl)methyl)phenyl) ethyl acetate (**4**).

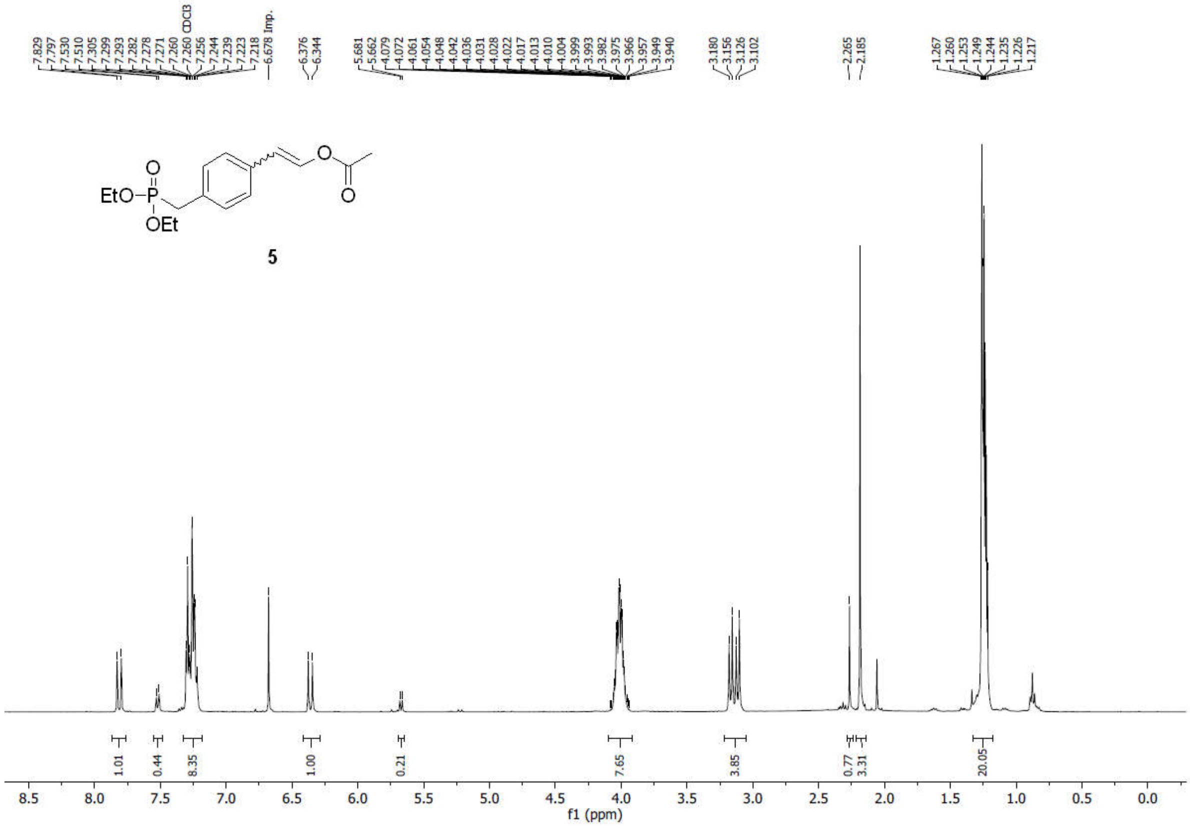

Figure S13. ^{1}H NMR (400 MHz, $CDCl_3$) of 4-((diethoxyphosphoryl)methyl)styryl acetate (**5**).

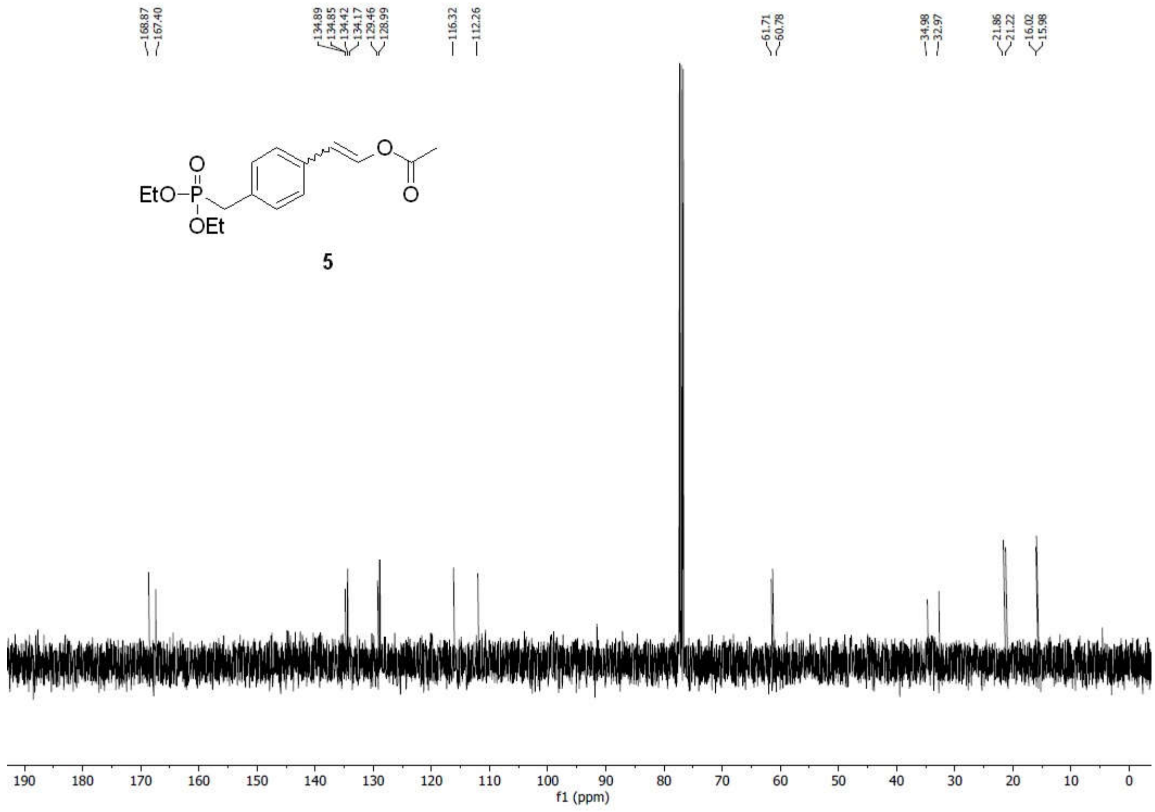

Figure S14. ^{13}C NMR (400 MHz, $CDCl_3$) of 4-((diethoxyphosphoryl)methyl)styryl acetate (**5**).

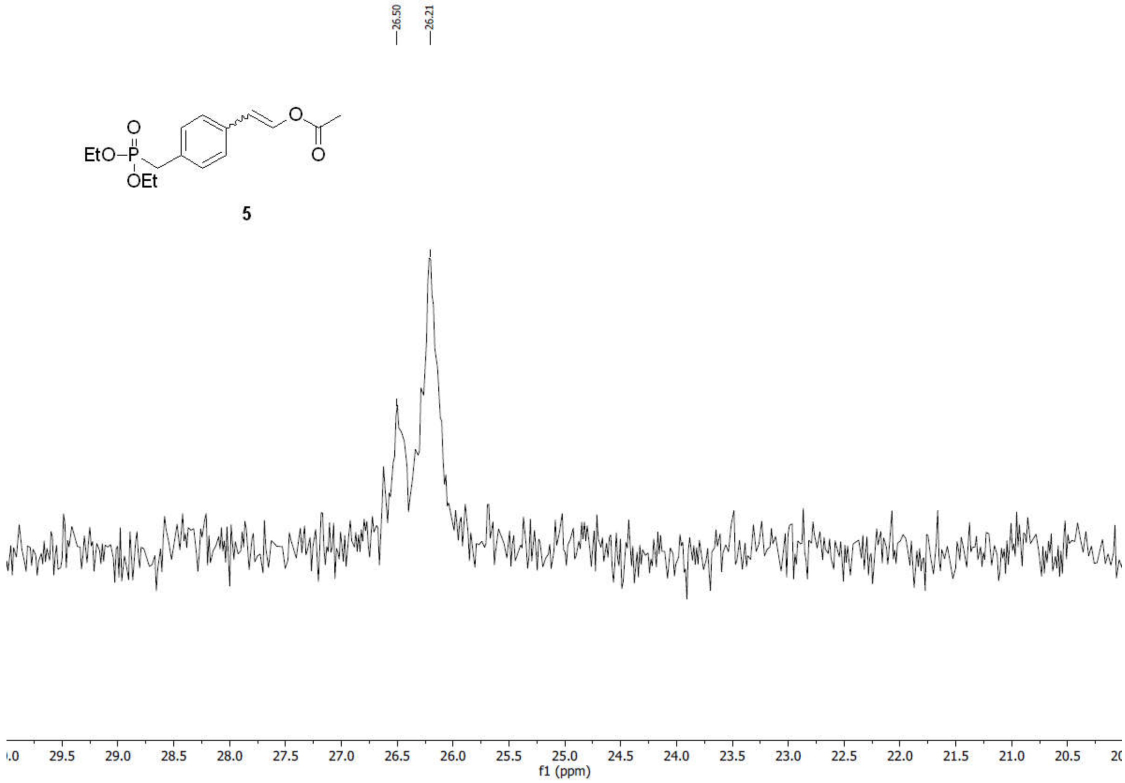

Figure S15. ^{31}P NMR (400 MHz, $CDCl_3$) of 4-((diethoxyphosphoryl)methyl)styryl acetate (**5**).

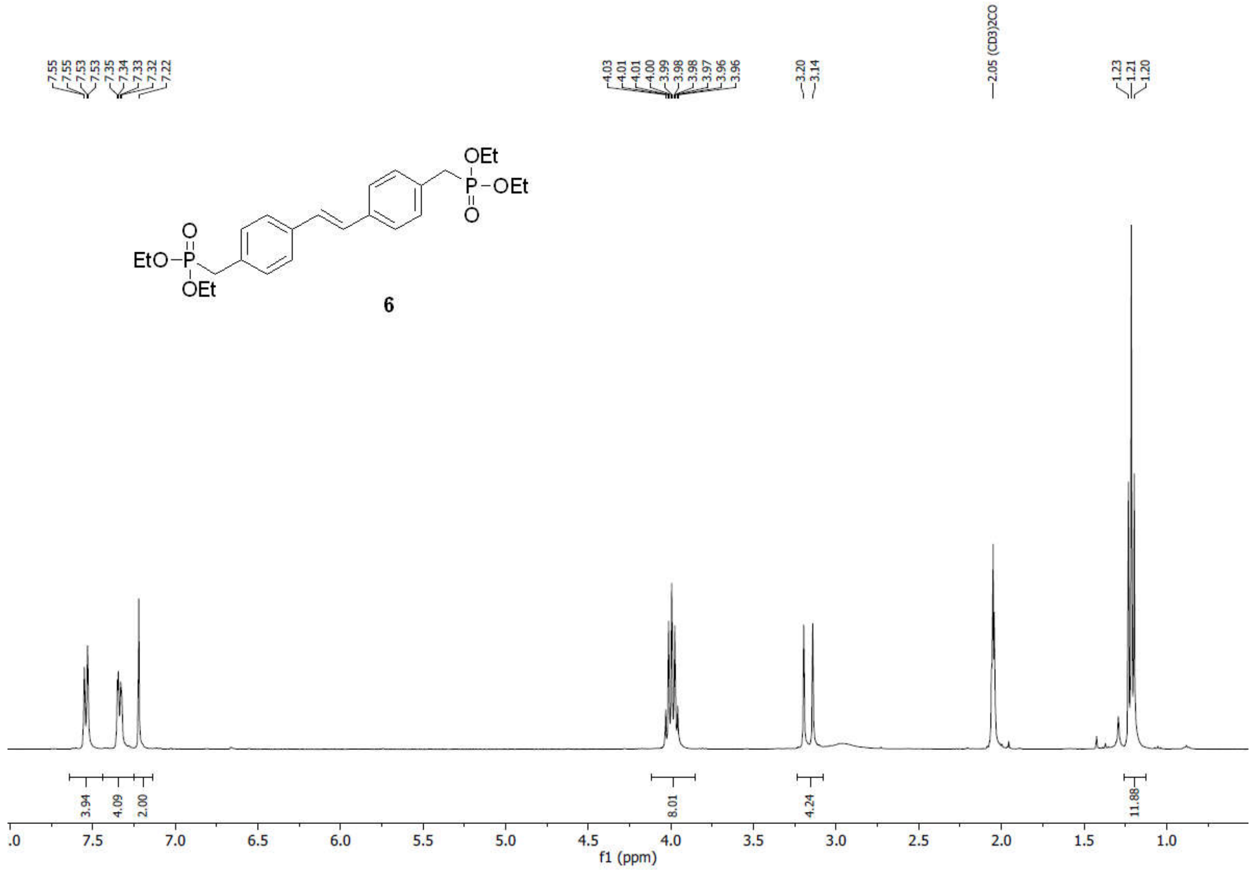

Figure S16. ^{1}H NMR (400 MHz, Acetone-d_6) of (E)-4,4'-bis(diethylphosphonatemethyl)stilbene (**6**).

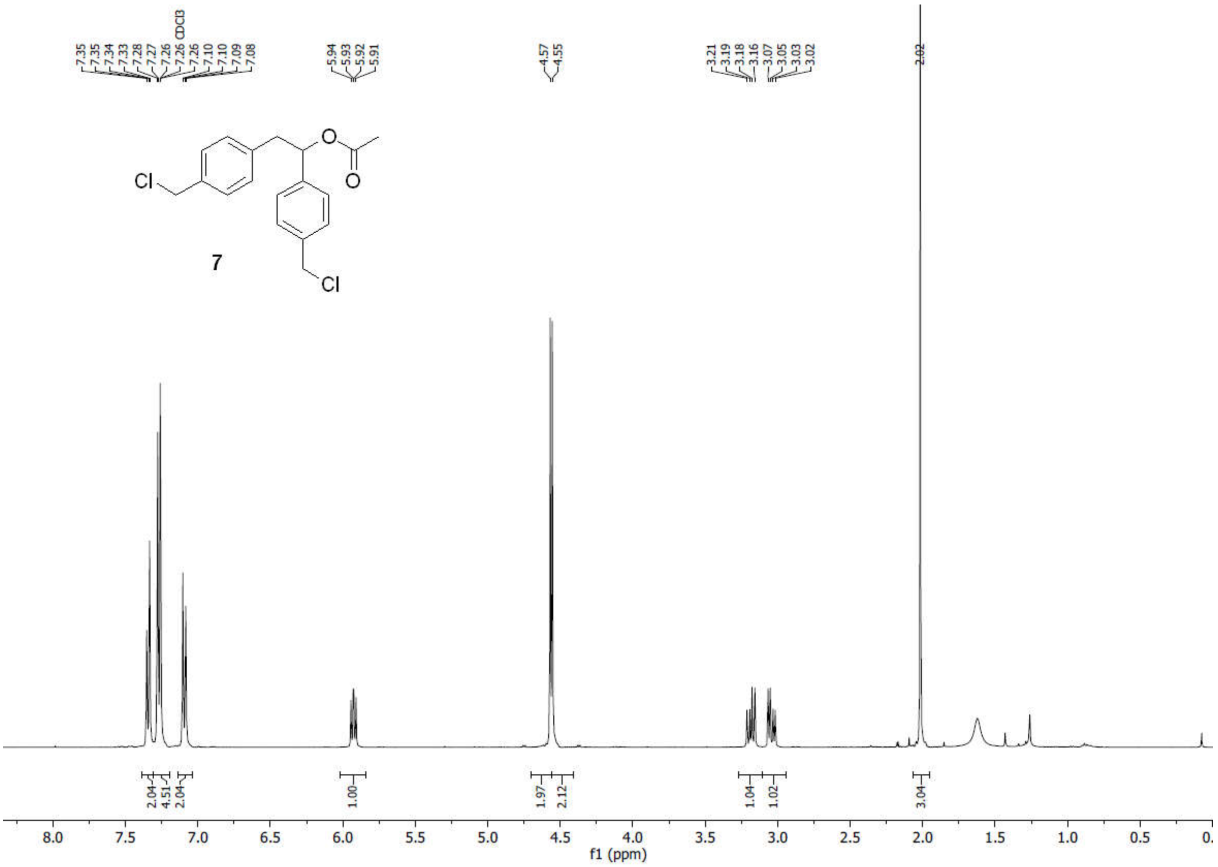

Figure S17. ^{1}H NMR (400 MHz, $CDCl_3$) of 1,2-bis(4-(chloromethyl)phenyl)ethyl acetate (**7**).

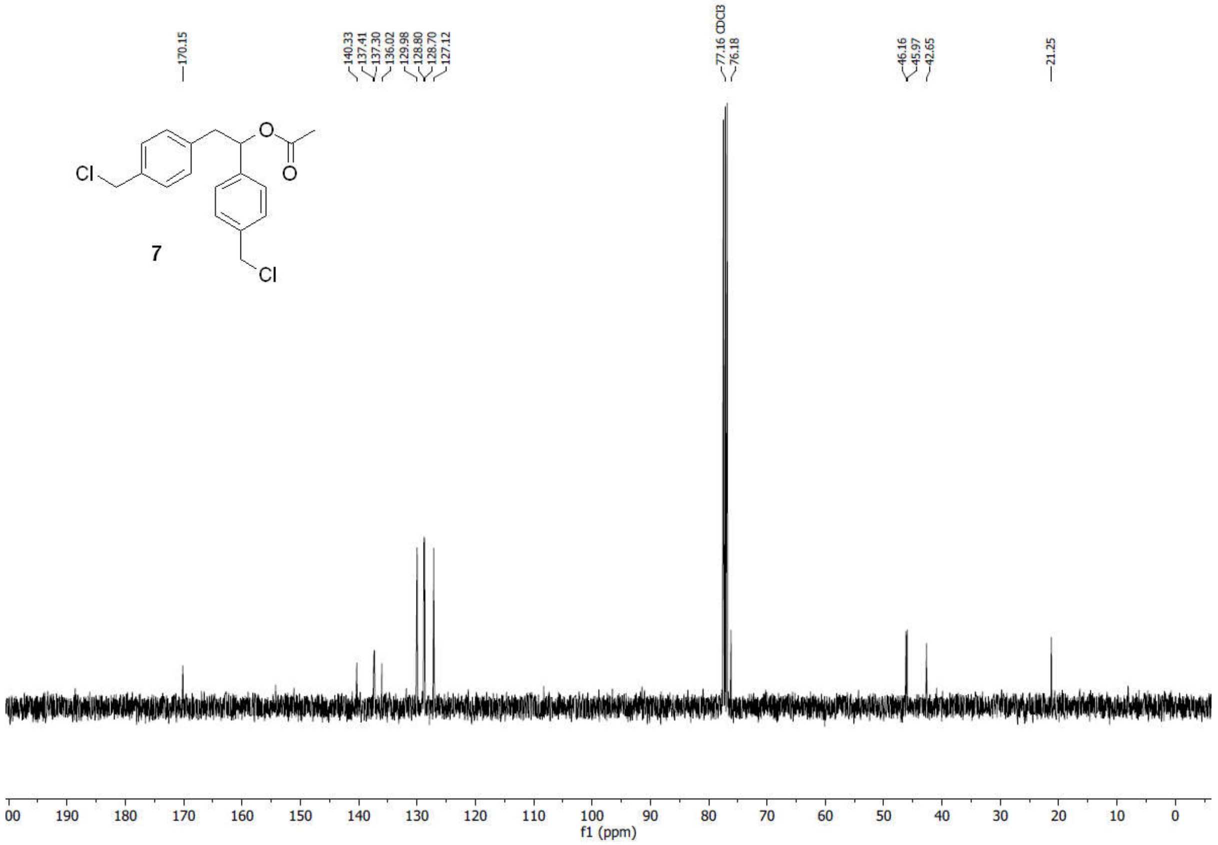

Figure S18. ^{13}C NMR (400 MHz, $CDCl_3$) of 1,2-bis(4-(chloromethyl)phenyl)ethyl acetate (**7**).

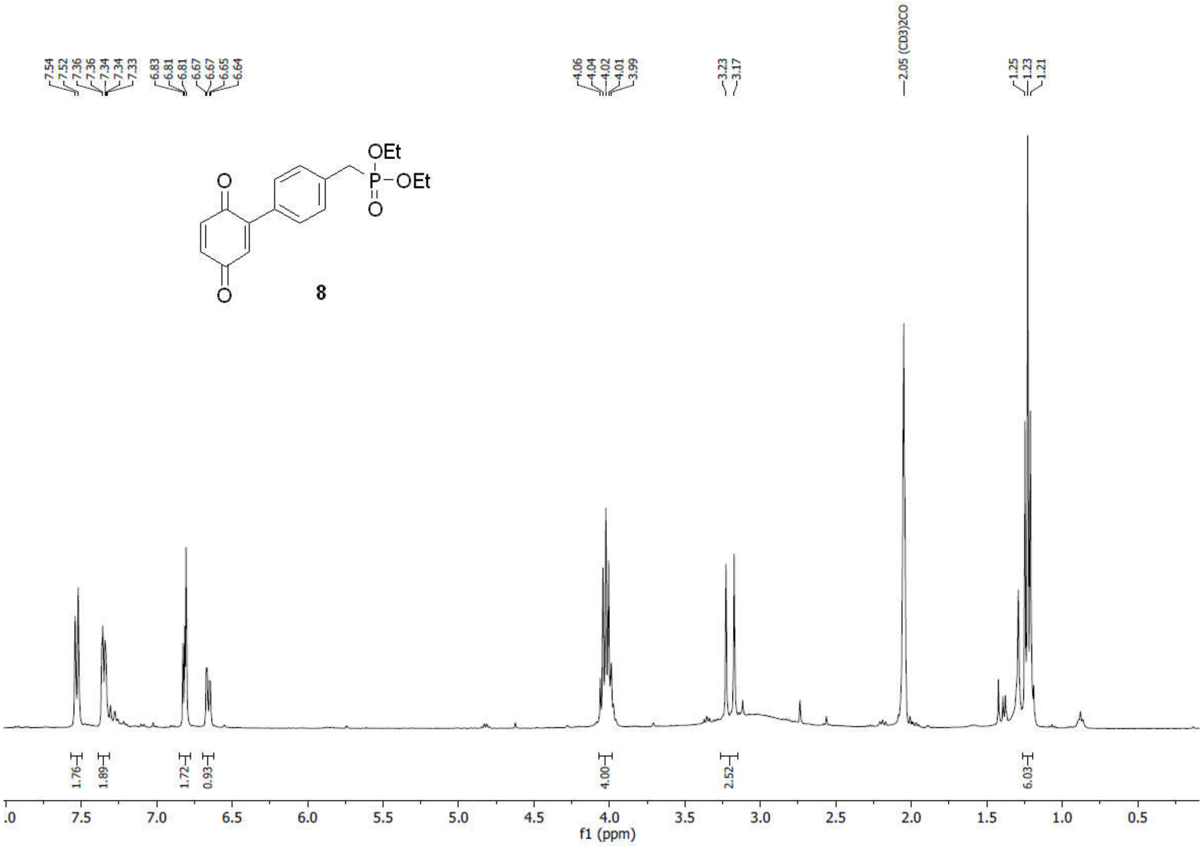

z, Acetone-d6) of diethyl ((2',5'-dioxo-2',5'-dihydro-[1,1'-biphenyl]-4-yl)methyl)phosphonate (**8**).

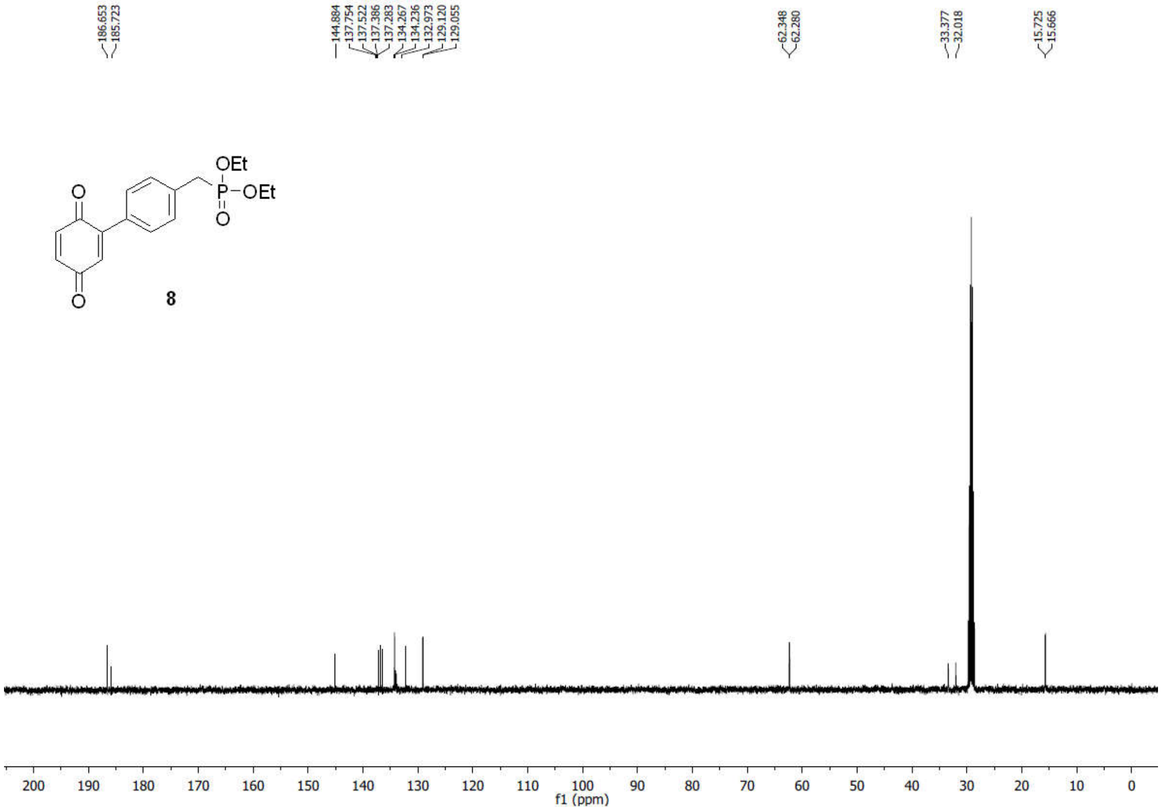

Figure S20. ^{13}C NMR (400 MHz, Acetone-d_6) of diethyl ((2',5'-dioxo-2',5'-dihydro-[1,1'-biphenyl]-4-yl)methyl)phosphonate (**8**).

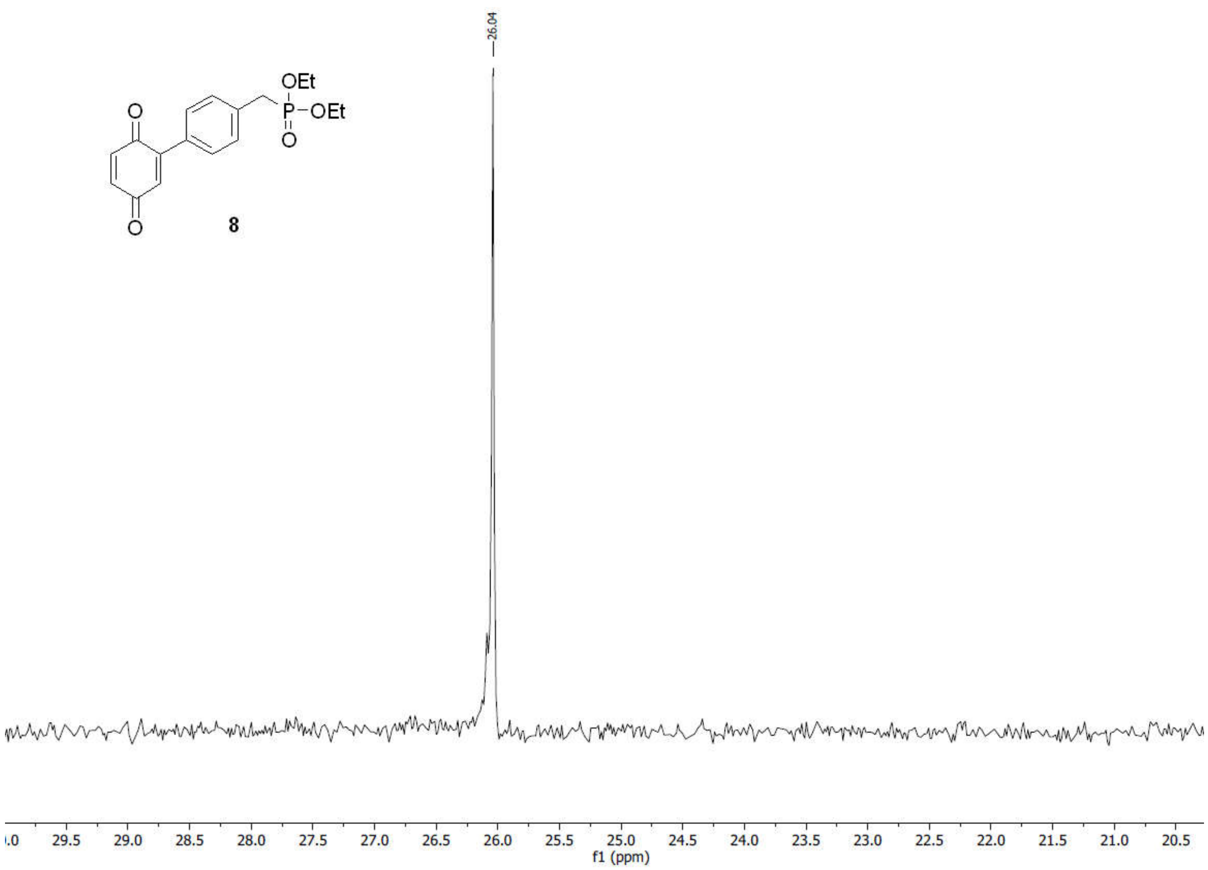

H
z, Acetone-d_6) of diethyl ((2',5'-dioxo-2',5'-dihydro-[1,1'-biphenyl]-4-yl)methyl)phosphonate (**8**).

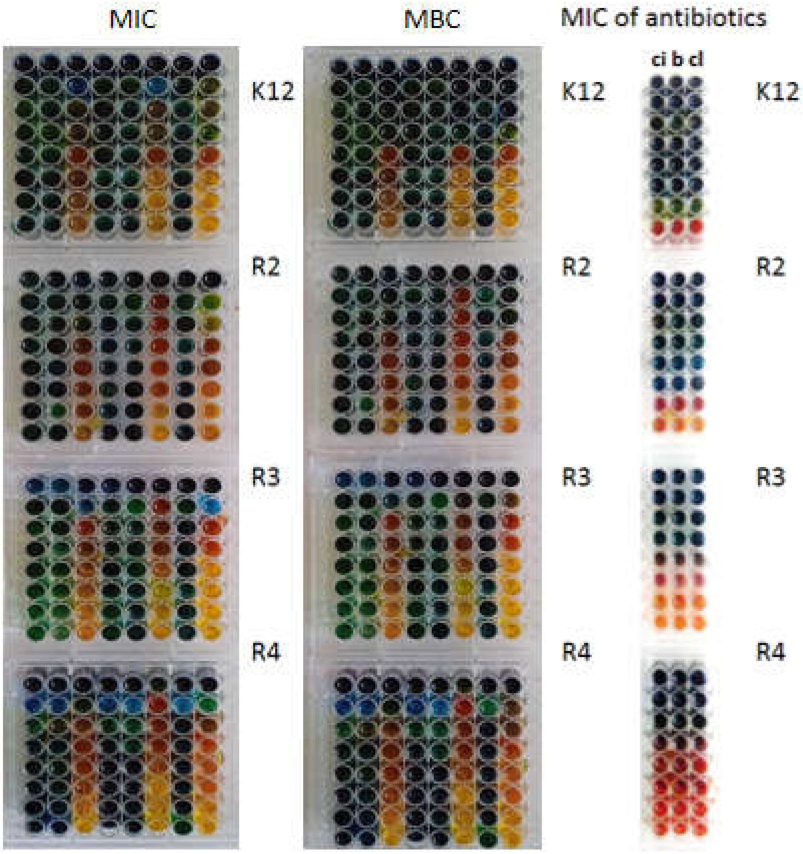

Figure S22. Examples of MIC and MBC on microplates with different concentration of studied compounds (μg/mL^{-1}). Resazurin was added as an indicator of microbial growth with K12, R2, R3, and R4 strains with tested 8 compounds, as described in Table 1. Additionally, examples of MIC with different strains K12, R2, R3, and R4 of studied antibiotics with ciprofloxacin (ci), bleomycin (b), and cloxacillin (cl) in (μg/mL).

Panel A

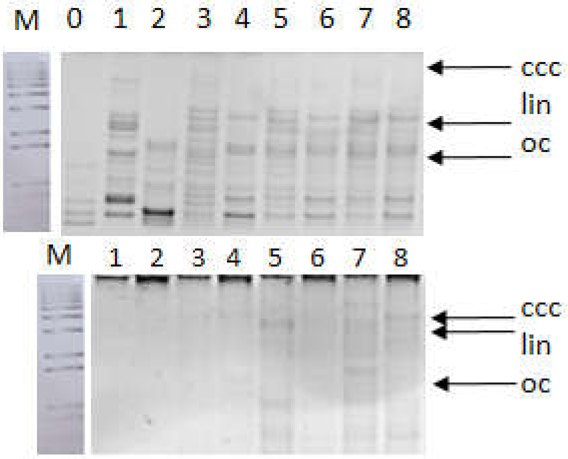

Panel B

Figure S23. An example of an agarose gel electrophoresis separation of isolated plasmids DNA on R4 strains modified with tested compounds (Panel A), as shown in Figure 3, and digested with repair Fpg protein (Panel B). M = marker.

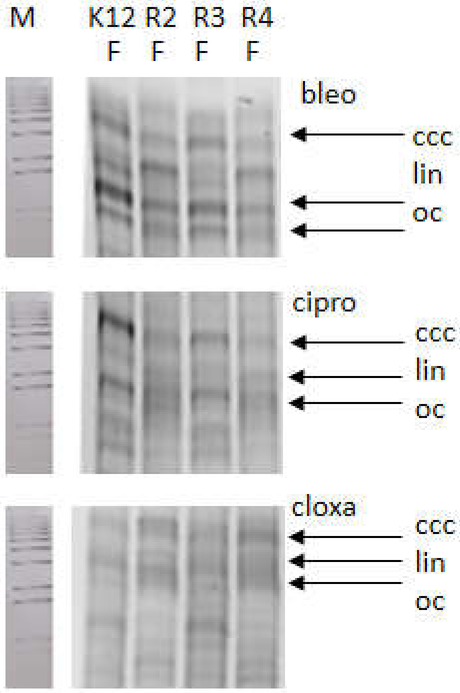

Figure S24. Example of an agarose gel electrophoresis separation of isolated plasmids DNA from K12 and R4 strains modified with antibiotics: bleomycin, ciprofloxacin, and cloxacillin digested (or not) with repair enzymes Fpg. M = marker.
